# Neural effects differ for learning highly iconic versus non-iconic signs in hearing adults

**DOI:** 10.1017/s1366728923000809

**Published:** 2023-11-23

**Authors:** Emily M. Akers, Katherine J. Midgley, Phillip J. Holcomb, Gabriela Meade, Karen Emmorey

**Affiliations:** 1 Department of Psychology, San Diego State University, San Diego, California, USA; 2 Department of Neurology, Mayo Clinic, Rochester, Minnesota, USA; 3 School of Speech, Language, and Hearing Sciences, San Diego State University, San Diego, California, USA

**Keywords:** event-related potentials, American Sign Language, iconicity, second language learning, N400

## Abstract

Little is known about the neural changes that accompany sign language learning by hearing adults. We used ERPs and a word-sign matching task to assess how learning impacted the N400 priming effect (reduced negativity for translations compared to unrelated trials). English monolinguals (N = 32) learned 100 ASL signs – half highly iconic (meaning was guessable), half non-iconic. In contrast to non-iconic signs, little learning was needed for the highly iconic signs as translation accuracy was similar pre- and post-learning. Prior to learning, an N400 priming effect was observed only for iconic signs. After learning, the size of the priming effect increased for non-iconic signs (replicating word learning studies) but decreased for iconic signs. For deaf ASL signers (N = 20), iconicity did not modulate the size of the N400 priming effect. We conclude that the impact of iconicity on lexico-semantic processing is reduced following learning, as signs are integrated into an emerging visual-manual lexicon.

## Introduction

Although the neurophysiological changes that occur with second language (L2) word learning for spoken languages are well documented (e.g., McLaughlin et al., 2004; Midgley et al., 2009; Pu et al., 2016; Yum et al., 2014), almost nothing is known about how the brain changes during L2 sign learning. Studies of L2 word learning using event-related potentials (ERPs) have targeted the N400, a negative-going wave that peaks ~400 ms after word onset and is thought to reflect lexico-semantic processing (Grainger & Holcomb, 2009). Modulations of the N400 response can occur in the very early stages of L2 vocabulary acquisition, even after only a few hours of L2 instruction (McLaughlin et al., 2004; Soskey et al., 2016; Yum et al., 2014). For example, after instruction L2 learners show a larger N400 for L2 pseudowords than real L2 words (McLaughlin et al., 2004) and the N400 amplitude increases for new L2 words with learning (Soskey et al., 2016). Further, the amplitude, time course, and/or scalp distribution of the N400 varies as a function of L2 proficiency (Midgley et al., 2009; Moreno & Kutas, 2005; Soskey et al., 2016). For example, the onset of N400 effects tends to be later and have a larger amplitude for less proficient L2 speakers (e.g., Moreno & Kutas, 2005; Yang et al., 2018). These findings point to the N400 as a sensitive measure for tracking the acquisition of new linguistic knowledge and for assessing engagement of lexico-semantic processes in L2 word learning.

**Figure 1.**
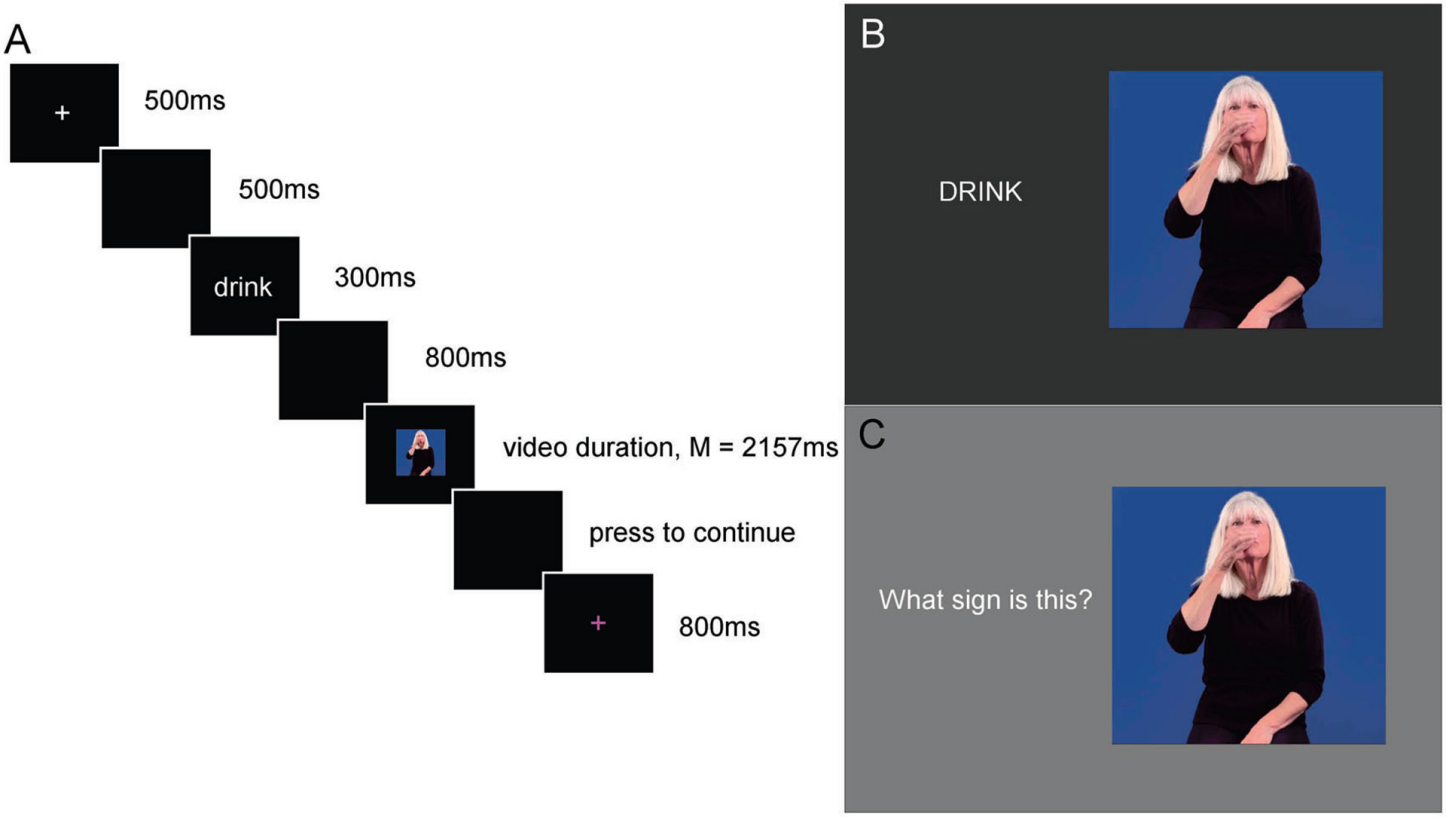
**A)** Schematic of a typical trial in the translation priming ERP session. **B)** Typical trial in the learning task. The English translation was presented simultaneously with a video of the ASL sign. **C)** Typical trial in the test section of learning task. Participants gave their answer verbally to the experimenter in the room.

Most ERP studies of L2 learning are conducted with unimodal bilinguals who acquire L2 vocabulary from a language that is in the same modality as their first language (L1), i.e., participants with a spoken native language learn words from another spoken language. It is unknown whether or how the N400 changes with learning when speakers of a spoken language acquire new vocabulary from a signed language. Learning L2 signs differs from learning L2 words along several dimensions. First, signs and words are produced with different articulators (the hands vs. the vocal tract) and are comprehended by different primary perceptual systems (vision vs. audition). Second, signs are more likely to have a motivated mapping between their form and meaning compared to words, and these iconic signs are learned faster and more accurately than non-iconic signs (e.g., Campbell et al., 1992; Lieberth & Gamble, 1991; Morett, 2015; see Ortega, 2017, for review). Third, manual gestures produced by speakers can be considered “manual cognates” for the iconic signs that they resemble (Ortega et al., 2020). For example, the ASL sign DRINK^1^ (see Figure 1B in methods) resembles a typical pantomimic gesture for ‘drinking’, and the meaning of this highly iconic sign is transparent to non-signers, i.e., they can correctly guess the meaning of the sign (Sehyr & Emmorey, 2019).

Ortega et al. (2020) recently used ERPs to investigate how the neural response changes during learning for iconic signs from Sign Language of the Netherlands (NGT) that had either high or low overlap with gestures that are typically produced for the same concepts. For example, the gesture for ‘telephone’ looks very similar to the NGT sign for telephone (high overlap), whereas the gesture for ‘toothbrush’ (grasping the handle as if using a toothbrush) has less form overlap with the NGT sign (an extended index finger represents the toothbrush). In pre- and post-learning blocks, hearing L1 speakers of Dutch viewed a Dutch word followed by the NGT sign translation. The word-sign translation pairs were viewed passively (no task was performed). Upon first exposure, iconic signs that had low overlap with gesture elicited an enhanced P3 compared to signs with high overlap, but the effect disappeared after learning. The P3 component is known to index novelty effects, with a larger P3 for an unexpected stimulus (Friedman et al., 2001). Ortega et al. (2020) interpreted the enhanced P3 amplitude for low vs. high overlap signs as indicating that hearing adults used their implicit knowledge of gestures to anticipate the form of signs, which matched expectations for the high, but not low overlap signs. After learning, participants were able to generate appropriate expectations for both types of iconic signs. However, Ortega et al. (2020) observed no N400 effects of high vs. low gesture overlap, either before or after learning, perhaps because all the signs were iconic and therefore elicited meaning. Since both types of signs may have produced similar meaning-related activation, there would be no difference in the N400 response between the two conditions.

Mott et al. (2020) examined the impact of iconicity on the N400 component when hearing English speakers learned iconic and non-iconic signs from American Sign Language (ASL). Over three sessions, participants learned the English translations of iconic signs (mean iconicity rating = 5.2) and non-iconic signs (mean iconicity rating = 1.2); iconicity ratings were from hearing non-signers using a 7-point Likert scale (1 = not iconic at all; 7 = very iconic) and were retrieved from the ASL-LEX database (Caselli et al., 2017; Sehyr et al., 2021). The behavioral data indicated that the advantage for iconic signs (faster RTs and accuracy in a forced-choice translation task) decreased across the three training sessions. However, Mott et al. (2020) did not track the neural changes that occurred with learning. The neural response to iconic versus non-iconic signs was only measured after learning, using a word-sign matching task and comparing N400 translation priming effects for target signs in the translation versus unrelated word-sign pairs. The results revealed earlier and larger N400 priming effects for iconic than non-iconic signs, suggesting that iconicity speeds lexical access for L2 learners and that meanings of iconic signs are more robustly represented in an emerging lexicon. In contrast to hearing L2 learners, deaf adults who learned ASL from birth or during early childhood showed no effects of iconicity within the N400 time window. Thus, iconicity facilitates lexical access and lexico-semantic processing for new learners, but not for proficient signers. Emmorey et al. (2020) also found that iconicity did not modulate neural activity during lexical access for deaf signers.

## The current study

We build on the results from Ortega et al. (2020) and Mott et al. (2020) by investigating how the neural dynamics of sign recognition change with learning when highly iconic signs with guessable meanings (akin to manual cognates) and non-iconic signs become integrated into an emerging sign language lexicon. Hearing native English speakers were taught a total of 100 signs (half highly iconic, half non-iconic) and performed a word-sign matching task prior to and after learning while ERPs were recorded. A group of deaf proficient ASL signers performed the same task to provide a benchmark comparison for the effects of iconicity on priming observed in the beginning ASL learners.

We predicted an interaction between iconicity and learning. Prior to learning, we anticipated that highly iconic signs would elicit a significantly larger N400 priming effect (i.e., reduced negativity for matching word-sign pairs than for no-match pairs) compared to non-iconic signs. Whereas learners can anticipate the forms of highly iconic signs based on their gestural schemas for the English translation prime, this is much less likely for the non-iconic signs. After learning, it was not clear a priori how the N400 priming effects for the two types of signs would compare. The size of the N400 priming effect for the non-iconic signs should increase relative to the pre-learning session because meanings have become associated with these forms. If the N400 priming effect for iconic signs also increases after learning, this will suggest that the iconic and non-iconic signs are similarly integrated into an emerging ASL lexicon. That is, an increased N400 priming effect post-learning indexes lexical integration for L2 learners, even for highly iconic signs with transparent meanings. On the other hand, if the N400 priming effect is reduced for iconic signs after learning, this will suggest that increasing proficiency and an emerging L2 lexicon reduces the impact of iconicity on lexical access and lexico-semantic processing.

In addition, we predict that sign iconicity will not modulate the N400 priming effect for deaf signers, given the results of Mott et al. (2020) and Emmorey et al. (2020). On the other hand, it is possible that the type of iconicity targeted by this study (i.e., highly transparent signs that often correspond to common gestures) may impact how robustly perceptual and motoric semantic features are represented in the lexicon of proficient deaf signers (see Navarrete et al., 2017). In this case, we might find that iconic signs elicit a larger N400 priming effect compared to non-iconic signs in deaf signers.

## Methods

This project was preregistered. Preregistration information (https://osf.io/r3h6d), as well as data and materials (https://osf.io/vpzxs/), are available at the project’s Open Science Framework (OSF) page.

### Participants

Participants in the learning group were 32 hearing English-speaking adults with no prior exposure to ASL (18 females; mean age 21 years, SD = 2.37, range = 18–29 years). All participants were right-handed and had either normal or corrected-to-normal vision. Participants reported no history of neurological disorders or learning impairments. They were all recruited from San Diego State University and the surrounding area.

Participants in the signer group were 20 deaf ASL signers, all of whom became deaf before or at two years old (8 females; mean age 34 years, SD = 10.04, range = 20–54 years). Twelve participants were native signers (born into signing families), eight participants were early signers (acquired ASL prior to age six), and all reported ASL as their preferred language. Seventeen participants were right-handed, one was one left-handed, and two reported being ambidextrous. Two participants had a cochlear implant, and the external sound processor was removed before collecting data. All deaf participants had either normal or corrected-to-normal vision and reported no history of neurological disorders or learning impairments.

All participants were treated in accordance with SDSU IRB guidelines. They provided informed consent and were given monetary compensation for their participation.

### Stimuli

The stimuli consisted of 100 video clips of ASL signs produced by a native female signer. Videos were presented on an LCD video monitor while the participants sat 110 cm (43 in) away from the screen. The video size was 10 x 13.25 cm in the center of the screen with a visual angle of 5.21 x 6.89 degrees. The signer was positioned in the middle of the frame so that her signing could be perceived without the participant needing to move their eyes. Note that even novice ASL learners look at the signer’s face and do not track the hands (Emmorey et al., 2009). All videos started with the sign model in a resting position with her hands on her lap and ended when her hands returned to her lap. The average video length was 2157 ms (SD = 290 ms), with an average sign onset of 578 ms (SD = 104 ms). Sign onset was determined as in Caselli et al. (2017). Briefly, sign onset is the first video frame in which the fully formed handshape contacts the body, and if the sign does not have body contact, then onset is defined as the first video frame in which the fully formed handshape arrives at the target location near the body or in neutral space before starting the sign movement. Naïve learners are not expected to be sensitive to information within the transition to sign onset and should only show priming effects after sign onset.

Fifty highly iconic signs were selected based on ratings from the ASL-LEX database (http://asl-lex.org; Caselli et al., 2017; Sehyr et al., 2021). Iconicity ratings were completed by hearing non-signers using a scale of 1 (not iconic) to 7 (very iconic). The iconic signs all had ratings over 5.0 (M = 6.3, SD = .51). In addition, to help ensure that the meanings of iconic signs were relatively transparent or “guessable”, we utilized the transparency ratings from the ASL-LEX database and collected additional ratings when transparency information was not available in the database. To gather transparency ratings, American hearing non-signers were asked to guess the meaning of an ASL sign and then to rate how obvious their guessed meaning would be to others, on a scale of 1 (not obvious at all) to 7 (very obvious). All iconic signs had a transparency rating of over 4.0 (M = 5.05, SD = .60). Examples of highly iconic, transparent signs are CIRCLE (index finger traces a circle in the air) and BRUSH (depicts brushing one’s hair); video links for all signs are on the project’s OSF page in the stimuli document (https://osf.io/vpzxs/). The average video length for the iconic signs was 2189 ms (SD = 331 ms), and the average sign onset within the video was 569 ms (SD = 97 ms).

The other 50 signs^2^ were non-iconic with an average video length of 2124 ms (SD = 242 ms), and an average sign onset within the video of 587 ms (SD = 111 ms). These signs had iconicity ratings under 3.0 (M = 1.92, SD = .47) and transparency ratings under 4.0 (M = 3.37, SD = .34). The iconic and non-iconic signs were also matched on ASL sign frequency (based on ratings from ASL-LEX), word frequency of their English translations (Van Heuven et al., 2014), concreteness of their English translations (Brysbaert et al., 2014), number of hands used in the sign, length of video, and average sign onset. Table 1 provides descriptive statistics for the sign stimuli.

## Procedure

### ERP Sessions

There were two ERP sessions, one before any learning took place and one after two learning sessions. Each ERP session began with a ‘grooming’ gesture detection task in which participants passively viewed all the 100 signs and pressed a button on a game pad when they detected an occasional grooming gesture (e.g., when the model scratched her head). This study was designed to investigate the neural response to iconic and non-iconic signs without requiring a meaning decision, and the results of this task will be reported separately. Participants then performed a translation matching task in which they saw an English word (the prime) followed by an ASL sign (the target) and were asked to decide whether the meaning of the word matched the meaning of the ASL sign, pressing one button on the game pad for “yes” and another for “no”. In the match condition, each sign was preceded by its English translation (e.g., *bowling*-BOWLING). In the no-match condition, the signs were preceded by the English translation of another learned sign (e.g., *triangle*-BOWLING) from the same condition (e.g., both TRIANGLE and BOWLING are iconic signs). Thus, all words were presented once in each of the two conditions. The response buttons were counterbalanced across participants. Match and no-match trials were pseudo-randomly intermixed (i.e., no more than three trials in a row from the same condition).

Each trial began with a white fixation cross for 500 ms followed by a blank screen for 500 ms. Immediately after the blank screen an English prime word was displayed in the center of the screen for 300 ms. Following the prime word was an 800 ms blank screen, and then the target video of a sign would play. Immediately after each sign target a blank screen would appear until the participant pressed a button indicating their match/no-match decision. Participants were not given feedback about whether their response was correct or not. After the button press a trial-ending 800 ms purple fixation was displayed indicating it was OK to blink before the beginning of the next trial (see Figure 1A for a schematic of a typical trial). Participants were asked to respond as quickly and as accurately as they could. For the recording session before learning took place, participants were asked to make their best guess as to whether the English word and the ASL sign matched in meaning or not. For the deaf signers and after learning for the hearing participants, the instructions were to decide whether the English word and ASL sign were translations.

In each ERP session learners saw one of two lists, A and B, both of which contained the same signs (100 match and 100 no-match trials), but in different orders. Whether list A or list B was presented before learning was counterbalanced across participants. The lists were also counterbalanced across the deaf participants (who only received one ERP session and thus only one list). Six additional signs (3 iconic and 3 non-iconic) were learned and were used in a short practice session for the word-sign matching task prior to each ERP session to get the participants used to the task and to provide time for any questions. These trials were not included in the analyses.

### Learning sessions

Hearing participants received two learning sessions and four testing sessions. After the first ERP session in which participants guessed whether an English word matched an ASL sign, participants were brought into a new room to learn all the signs they had just seen. They were shown a PowerPoint slideshow where a video of each sign was presented next to its English translation (see Figure 1B for a typical learning trial). Participants were able to replay the videos of the signs, and they were told to try to remember each sign using whatever strategy they wished.

After participants had watched all the signs with their English translations, they were tested. Participants watched another PowerPoint with the signs in a different order and without the English words. For each sign, they were instructed to tell a researcher in the room what they thought the English translation was (see Figure 1C for a typical test trial). The researcher gave participants feedback and, if wrong, told them the correct English translation. This testing session was completed on the first day of the study (after the ERP recording session).

In the second learning session (one or two days after the first ERP/learning session), participants met with the researcher via Zoom. They were tested again to see how much they remembered from the previous session using the same testing format with the researcher’s screen shared with the participant. After this second testing session, participants undertook another learning session where they saw all the signs again with their English translations. Then they were tested for a third time.

On the third session (one to two days after the second learning session), after completing the ERP session (the word-sign matching task), participants were tested one last time to assess how well they remembered all the signs.

For each learning and testing session, the order of signs in the PowerPoint presentation was pseudo-randomized, such that no more than four iconic or non-iconic signs were in a consecutive order. Participants were told not to practice any of the signs outside of the experiment sessions during the week of testing.

### EEG recording

All participants were seated in a comfortable chair in a darkened, sound attenuating room. EEG was continuously recorded through a 29-channel cap with tin electrodes (Electro-cap International, Inc., Eaton, OH). There were four loose electrodes placed on the participant’s head at the following locations: one underneath the left eye to track blinking, one on the side of the right eye to track horizontal eye movements, and one placed on each mastoid bone behind the ear – the left mastoid was used as the reference electrode. All electrodes were connected using a saline-based gel (Electro-Gel), and impedances were reduced to under 2.5kΩ. The data was collected through Curry Data Acquisition software with a sampling rate of 500 Hz, and the EEG signal was amplified by a SynAmpsRT amplifier (Neuroscan-Compumedics, Charlotte, NC) with a bandpass of DC to 100 Hz.

ERPs were time-locked offline to the onset of the target ASL sign (video onset) with a 100 ms pre-stimulus baseline. ERPs from individual sites were processed with a 15 Hz low-pass filter prior to analysis. Trials that had artifact from eye movements or drifty electrodes within 1500 ms of target onset were removed from analysis (9.5 trials, 4.6% on average).

### Data analysis: word-sign matching task

Reaction times (RTs) and accuracy were measured for the forced-choice English-ASL matching task that was performed while ERPs were recorded before and after learning. RTs were measured from video onset to when the participant made their button press response.

For the analysis of the ERP data, following Mott et al. (2020), all trials (correct and incorrect) were included in the analyses, ERPs were time-locked to video onset, and nine electrode sites were analyzed to identify effects across a representative sample of scalp sites (see Supplementary Figure 1 for the sites analyzed). Prior language learning studies in our lab have shown that this grid analysis approach offers the best coverage of the scalp with the fewest number of statistical comparisons (e.g., Yum et al., 2014). Also following Mott et al. (2020), we quantified the ERP data in four windows: 400–600 ms, 600–800 ms, 800–1000 ms, and 1000–1400 ms (baselined to the mean amplitude in the 100 ms pre-stimulus epoch). Since we time-locked to video onset, and the average sign onset occurred at 578 ms, we expected N400 effects to be most notable in the 800–1000 ms time-window. Preliminary analyses indicated that the main effect of Relatedness (match vs. no-match) was significant in all four epochs in both hearing learners and deaf signers.

Because our primary research questions were related to changes in the size of the priming effects for iconic compared to non-iconic signs and to reduce the complexity of the ANOVA design, we performed our analyses on the mean amplitude of difference waves (no-match minus match). This approach provides a direct measure of the size of the priming effects as opposed to the amplitudes in raw ERP waves (however, the raw ERPs are plotted in the Figures). Thus, a within-subjects design ANOVA was used to assess the size of priming effects as a function of Learning (before learning vs. after learning), Iconicity (iconic vs. non-iconic), and two scalp distribution factors; Anteriority of electrode site on the scalp (frontal, central, parietal), and Laterality of electrode site on the scalp (left, middle, right). Since the participants saw the same signs before and after learning and the iconic and non-iconic signs were matched on a number of variables (see Table 1), we looked specifically for main effects of Iconicity, Learning, and the interactions between these two variables. Because we hypothesized there would be differences in the effects of learning on the pattern of priming for iconic and non-iconic signs, separate planned follow-up analyses were conducted for these two types of signs, but only when the Learning x Iconicity effect was significant in the main omnibus ANOVA.

Finally, in order to track the subtle differences in priming onset between the deaf signers and hearing learners, we also conducted a time-course analysis at the Cz electrode site in eight adjacent 50 ms intervals starting at 400 ms and going through to 800 ms. Because of the use of multiple analyses all *p*-values were FDR corrected (Groppe et al., 2011).

Significant results (*p* < .05) are reported below. Partial eta squared ($\eta_{p}^{2}$) is reported as a measure of effect size, and Greenhouse-Geisser (Greenhouse & Geisser, 1959) correction was used for all significant effects with a degree of freedom numerator greater than one.

## Behavioral results

### Learning tests

There was a total of four learning test scores for each participant: 1) after the first learning session, 2) prior to the second Zoom learning session, 3) after the second Zoom learning session, and 4) after the second (post-learning) ERP session. Accurate answers were based on whether the participant provided the correct English translation for a given ASL sign. Differences in tense did not affect the accuracy, e.g., jumping for jump. Mean accuracy and standard deviations for each test session are given in Table 2.

ANOVAs were performed on percent correct (arc sine transformed) for all four testing sessions. There were significant main effects of Test Session (F(3,93) = 42.84, *p* < .0001, $\eta_{p}^{2}$ = .2124) – accuracy improved with learning, and of Iconicity (F(1,31) = 133.72, *p* < .0001, $\eta_{p}^{2}$ = .2164) – accuracy was higher for iconic signs. There was also a significant interaction between Test Session and Iconicity (F(3,93) = 141.54, *p* < .0001, $\eta_{p}^{2}$ = .092) – accuracy between sessions improved more for non-iconic signs than for iconic signs.

### Word-sign matching task: hearing learners

**Figure 2.**
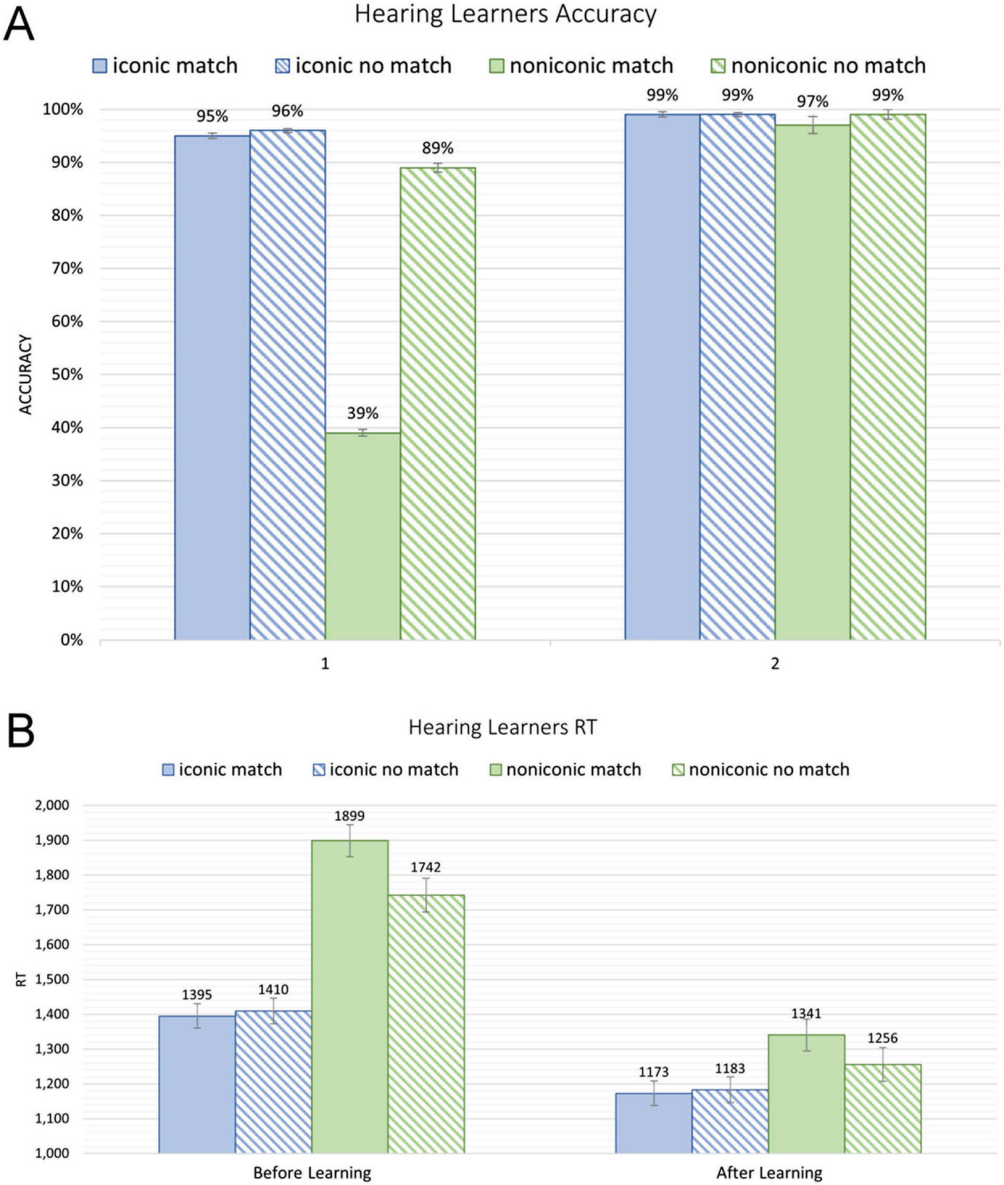
**A)** Hearing learners matching accuracy (and standard error bars) for the word-sign matching task. **B)** Hearing learners mean RTs in ms (and standard error bars) for the word-sign matching task.

Table 3 provides the means and standard deviations for accuracy and response times for the matching task for the hearing learners. ANOVAs were performed on the RTs and accuracy (arc sine transformed) for all trials. The accuracy results are illustrated in Figure 2A. Overall, participants were more accurate after learning, indicated by a significant main effect of Learning, F(1,31) = 856.46, *p* < .0001, $\eta_{p}^{2}$ = .2058). Participants were also less accurate in making match decisions, indicated by a significant main effect of Matching, F(1,31) = 151.28, *p* < .0001, $\eta_{p}^{2}$ = .1077). Participants were more accurate for iconic signs, indicated by a main effect of Iconicity, F(1,31) = 801.09, *p* < .0001, $\eta_{p}^{2}$ = .1623). There were also several significant two-way interactions that modulated these main effects. There was an interaction between Learning and Matching (F(1,31) = 117.67, *p* < .0001, $\eta_{p}^{2}$ = .0928) – accuracy increased more for match than for no-match trials after learning. There was an interaction between Learning and Iconicity (F(1,31) = 602.19, *p* < .0001, $\eta_{p}^{2}$ = .1353) – accuracy increased more for non-iconic than iconic signs as a function of learning. There was an interaction between Matching and Iconicity (F(1,31) = 141.65, *p* < .0001, = .095) – for non-iconic signs, participants were less accurate for match than no-match decisions, a difference that was not observed for iconic signs. Finally, there was a significant three-way interaction between Learning, Matching, and Iconicity (F(1,31) = 124.42, *p* < .0001, $\eta_{p}^{2}$ = .0835). As can be seen in Figure 2A, this three-way interaction was due to non-iconic signs showing the greatest improvement in accuracy for match decisions^3^.

The RT results are shown in Figure 2B. RTs were trimmed so that trials outside the typical range did not affect the results (> 2.5 standard deviations from the mean for all trials). There was a significant main effect of Learning (F(1, 31) = 74.38, *p* < .0001, $\eta_{p}^{2}$ = .2928) – participants were faster after learning, and there was a significant main effect of Matching (match vs. no-match conditions) (F(1,31) = 13.95, *p* = .0008, $\eta_{p}^{2}$ = .0062) – participants made slower decisions in the match condition compared to the no-match condition. There was also a significant main effect of Iconicity (F(1,31) = 284.8, *p* < .0001, $\eta_{p}^{2}$ = .1525) – participants responded faster for iconic signs compared to non-iconic signs. There were also several significant two-way interactions that modulated these main effects. Learning interacted with Iconicity (F(1,31) = 135.76, *p* < .0001, $\eta_{p}^{2}$ = .0465) – participants’ RTs were faster after learning, with a greater decrease for non-iconic signs (521 ms versus 224 ms). In addition, Matching interacted with Iconicity (F(1,31) = 57.47, *p* < .0001, $\eta_{p}^{2}$ = .0093) – for non-iconic signs, RTs were slower for match trials compared to no-match trials, but for iconic signs, there was no difference between match and no-match trials. There was also a three-way interaction between Learning, Matching, and Iconicity (F(1,31) = 6.51, *p* = .0159, $\eta_{p}^{2}$ = .0008). As can be seen in Figure 2B, RTs for match and no-match trials with iconic signs were similar before and after learning, but for non-iconic signs, the difference between match and no-match decisions decreased after learning.

### Word-sign matching task: deaf signers

Table 4 provides the means and standard deviations for RT and accuracy for the matching task for the deaf signers. For accuracy, there was no main effect of Iconicity or Matching, and no significant interaction between Iconicity and Matching (all *p*s > .10). For RTs, there was a significant main effect of Matching, F(1,19) = 8.88, *p* = .0077, $\eta_{p}^{2}$ = .0099 – RTs were faster for the match trials compared to the no-match decisions. There was no main effect of Iconicity and no interaction between Matching and Iconicity (all *p*s > .18).

### ERP results: hearing learners

**Figure 3.**
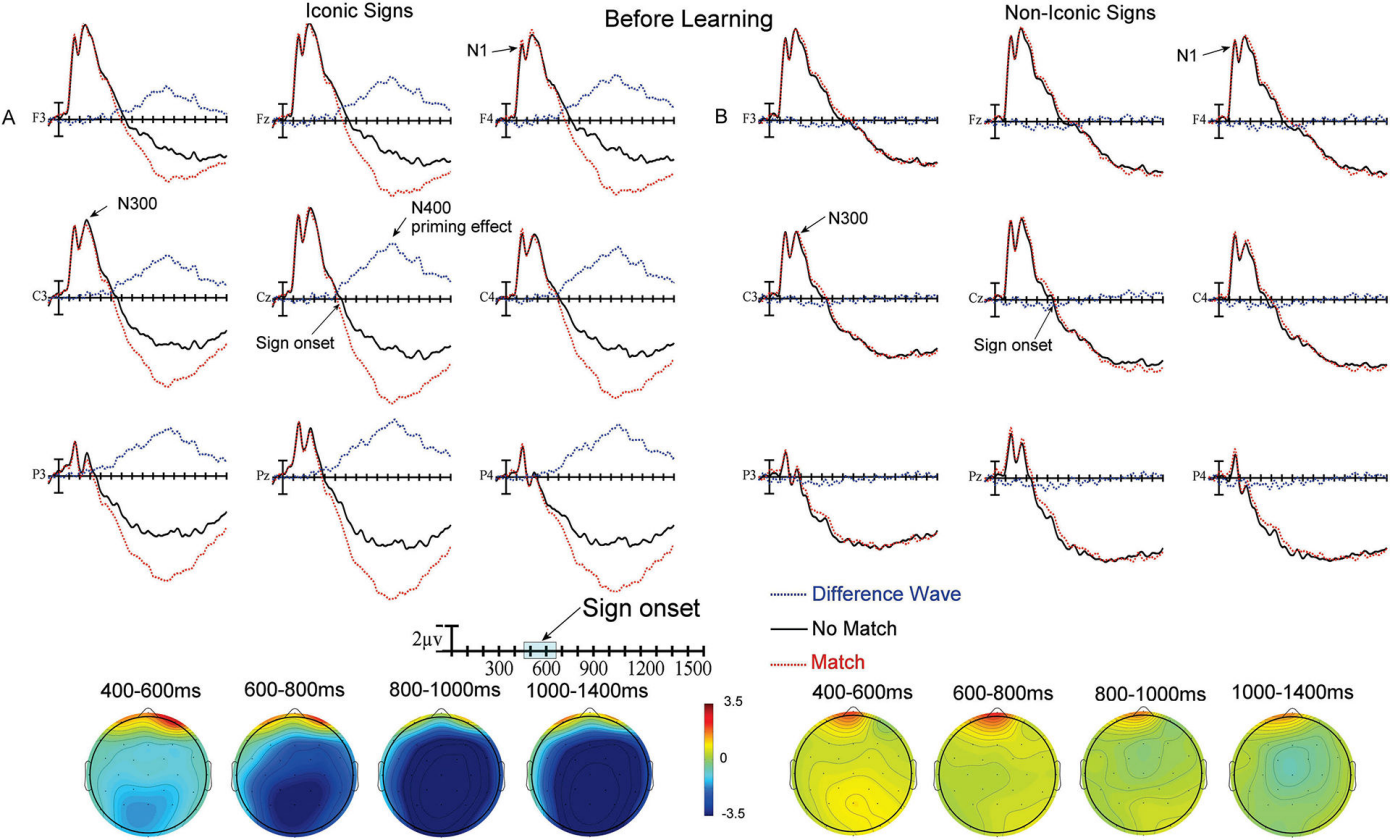
**A)** Top) ERPs to iconic ASL signs before learning at the 9 electrode sites used in the ANOVAs (Difference waves are No Match – Match trials). Negative is plotted up in this and all subsequent figures. Bottom) Voltage maps formed by subtracting no-match trial ERPs from match trial ERPs in the four latency ranges. **B)** Top) ERPs to non-iconic ASL signs before learning at the 9 electrode sites used in the ANOVAs (Difference waves are No Match – Match trials). Bottom) Voltage maps formed by subtracting no-match trial ERPs from match trial ERPs in the four latency ranges.

**Figure 4.**
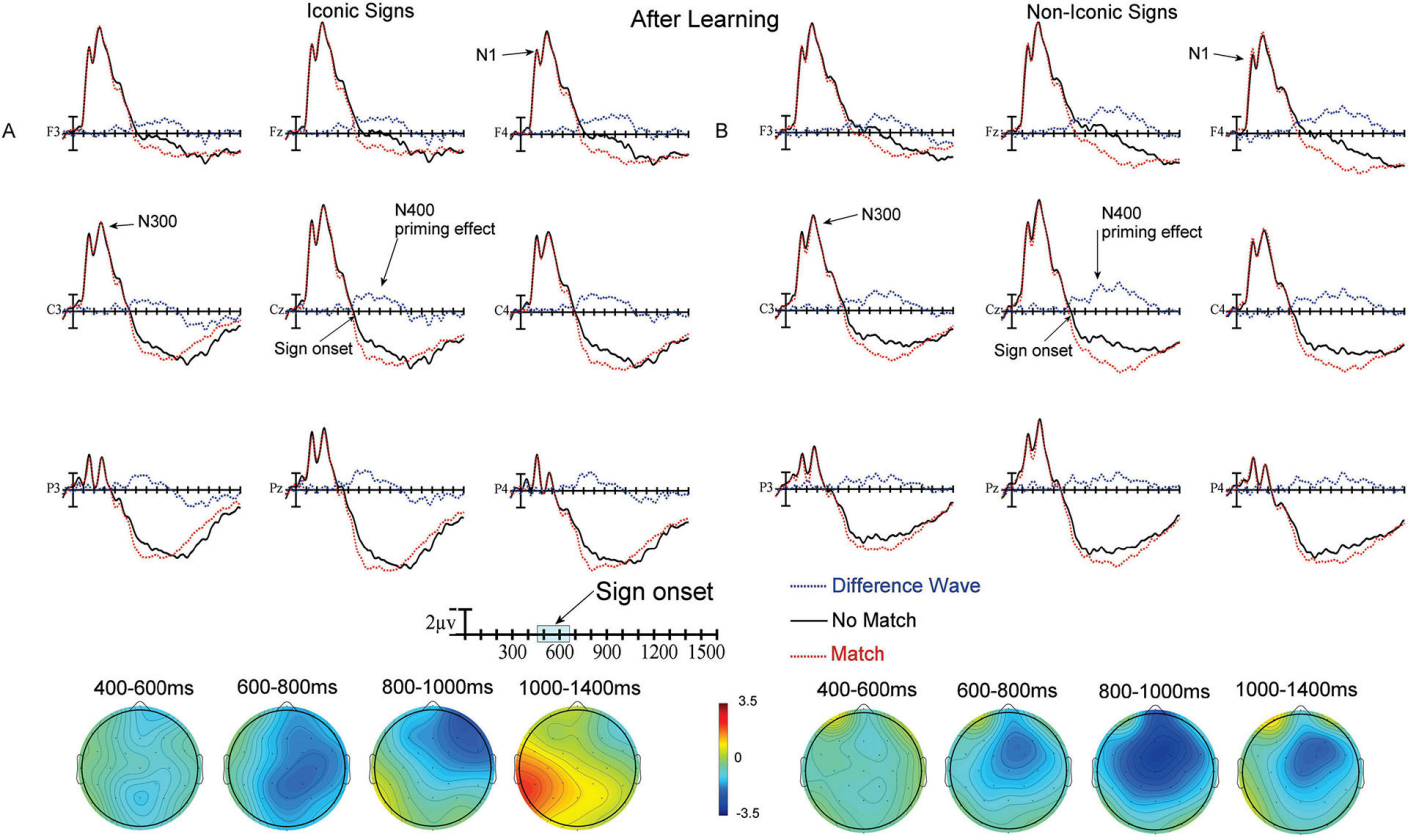
**A)** Top) ERPs to iconic ASL signs after learning at the 9 electrode sites used in the ANOVAs (Difference waves are No Match – Match trials). Bottom) Voltage maps formed by subtracting no-match trial ERPs from match trial ERPs in the four latency ranges. **B)** Top) ERPs to non-iconic ASL signs after learning at the 9 electrode sites used in the ANOVAs (Difference waves are No Match – Match trials). Bottom) Voltage maps formed by subtracting no-match trial ERPs from match trial ERPs in the four latency ranges.

Plotted in Figures 3 and 4 are the ERPs recorded to target signs in the matching translation task. The no-match (black) and match (red) ASL signs are overplotted for the iconic signs (Figures 3A and 4A, before and after learning) and for the non-iconic signs (Figures 3B and 4B, before and after learning). The difference waves (blue) resulting from subtracting match from no-match trials are also included since these are the waves quantified in the ANOVAs. As can be seen in all figures, there were very high amplitude early ERP components in the first 500 ms after the onset of the ASL video clips, including a widely distributed N1 peaking just before 200 ms and a following smaller positivity peaking just after 200 ms. These early ERP components were followed by a larger negativity (N300) peaking between 300 and 400 ms. After this point, there was a positive trend in the ERPs across the scalp for the remainder of the recording epoch. Finally, there were clear effects of priming (no-match vs. match) – as reflected in the departure from zero of the blue difference waves in the ERP in Figures 3 and 4, and the blue regions in the voltage maps.

### Hearing learners

#### 400–600 ms time epoch

For this first epoch, there was a main effect of Iconicity (F(1,31) = 5.02, *p* = .0323, $\eta_{p}^{2}$ = .0331), as well as a significant interaction between Learning and Iconicity (F(1,31) = 6.97, *p* = .0129, $\eta_{p}^{2}$ = .021) suggesting that the size of the priming effect (i.e., the amplitude of the difference waves) differed for iconic and non-iconic signs.

#### Planned interaction follow-up

During this epoch the amplitude of the difference waves to iconic signs did not differ as a function of Learning (before vs. after learning; all *p*s > .09, see Figures 3A and 4A difference waves and 400 – 600 ms voltage maps). However, there was a main effect of Learning for non-iconic signs (F(1,31) = 5.89, *p* = .0213, $\eta_{p}^{2}$ = .0745) – participants showed a significantly larger priming effect (more negative difference waves) after learning than they did before learning (see Figures 3B and 4B difference waves and 400 – 600 ms voltage maps).

#### 600–800 ms time epoch

During this second epoch, there was a main effect of Iconicity (F(1,31) = 9.25, *p* = .0048, $\eta_{p}^{2}$ = .064), as well as a significant interaction between Learning and Iconicity (F(1,31) = 18.98, *p* = .0001, $\eta_{p}^{2}$ = .0444), and between Learning, Iconicity, Laterality and Anteriority (F(4,124) = 3.67, *p* = .0094, $\eta_{p}^{2}$ = .0004).

#### Planned interaction follow-up

As in the previous epoch there were no significant differences in the size of the difference waves as a function of Learning for iconic signs (all *p*s > .07; see Figures 3A and 4A difference waves and 600 – 800 ms voltage maps), but there was a main effect of Learning for non-iconic signs (F(1,31) = 10.45, *p* = .0029, $\eta_{p}^{2}$ = .1136). However, the latter did not interact with either distributional factor. Participants showed a larger priming effect (larger difference waves) after learning than before for non-iconic signs (see Figures 3B and 4B difference waves and 600 – 800 ms voltage maps).

#### 800–1000 ms time epoch

There was a main effect of Iconicity (F(1,31) = 11.2, *p* = .0022, $\eta_{p}^{2}$ = .0569), as well as significant interactions between Learning and Iconicity (F(1,31) = 40.69, *p* < .0001, $\eta_{p}^{2}$ = .1218). Like the previous epoch, this latter effect also interacted with the two scalp distribution factors Learning, Iconicity, Laterality and Anteriority (F(4,124) = 3.43, *p* = .0106, $\eta_{p}^{2}$ = .0005).

#### Planned interaction follow-up

There was a main effect of Learning for iconic signs (F(1,31) = 21.21, *p* = .0001, $\eta_{p}^{2}$ = .1329), as well as an interaction between Learning and Laterality (F(2,62) = 3.64, *p* = .045, $\eta_{p}^{2}$ = .0009) and between Learning and Anteriority (F(2,62) = 10.77, *p* = .0013, $\eta_{p}^{2}$ = .0098). For iconic signs, participants showed a smaller priming effect after learning than they did before learning, and the difference was particularly evident at left hemisphere and posterior sites (see Figures 3A and 4A difference waves and 800 – 1000 ms voltage maps). There was also a main effect of Learning for non-iconic signs (F(1,31) = 13.27, *p* = .001, $\eta_{p}^{2}$ = .1413) – however, this effect was in the opposite direction to that found for iconic signs. For non-iconic signs, participants showed a larger priming effect (greater difference wave negativity) after compared to before learning (see Figures 3B and 4B difference waves and 800 – 1000 ms voltage maps).

#### 1000–1400 ms time epoch

In the final measurement epoch there was a main effect of Learning (F(1,31) = 10.87, *p* = .0025, $\eta_{p}^{2}$ = .0577), and interactions between Learning and Iconicity (F(1,31) = 47.55, *p* < .0001, $\eta_{p}^{2}$ = .139), Learning, Iconicity, and Laterality (F(2,62) = 6.53, *p* = .0038, $\eta_{p}^{2}$ = .0011), and between Learning, Iconicity, and Anteriority (F(2,61) = 4.19, *p* = .0425, $\eta_{p}^{2}$ = .0037).

#### Planned interaction follow-up

There was a main effect of Learning for iconic signs (F(1,31) = 38.95, *p* < .0001, $\eta_{p}^{2}$ = .278), as well as significant interactions between Learning and Laterality (F(2,62) = 4.62, *p* = .0171, $\eta_{p}^{2}$ = .0012), and Learning and Anteriority (F(2,62) = 12.6, *p* = .0006, $\eta_{p}^{2}$ = .0134). These interactions are due to greater difference wave negativity for iconic signs before learning compared to after learning, especially over more posterior and left hemisphere sites (see Figures 3A and 4A difference waves and 1000–1400 ms voltage maps). There was no main effect of Learning or interaction between Learning and scalp distribution for non-iconic signs; all *p*s > .11 (see Figures 3B and 4B difference waves and 1000–1400 ms voltage maps).

### ERP results: deaf signers

**Figure 5.**
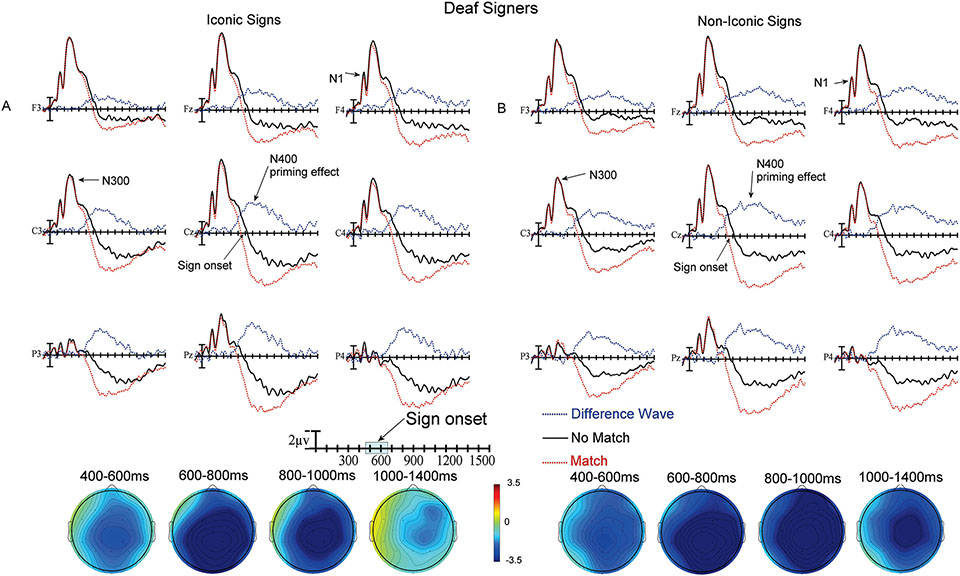
**A)** Top) ERPs to iconic ASL signs at the 9 electrode sites used in the ANOVAs (Difference waves are No Match – Match trials). Bottom) Voltage maps formed by subtracting no-match trial ERPs from match trial ERPs in the four latency ranges. **B)** Top) ERPs to non-iconic ASL signs at the 9 electrode sites used in the ANOVAs (Difference waves are No Match – Match trials). Bottom) Voltage maps formed by subtracting no-match trial ERPs from match trial ERPs in the four latency ranges.

The ERP results for the deaf signers are shown in Figure 5. Similar to the hearing learners, the early ERP components included a widely distributed N1 peaking just before 200 ms and a following smaller positivity peaking just after 200 ms. These early ERP components were followed by a larger negativity (N300) peaking between 300 and 400 ms. After this point, there was a positive trend in the ERPs across the scalp for the remainder of the recording epoch. In the analysis of the difference waves (i.e., the priming effect) there were no main effects of Iconicity or interactions between Iconicity and scalp distribution (either Laterality or Anteriority) in any of the epochs (all *p*s > .07). To verify that the large priming effects seen across epochs were indeed significant we also decided to look at the main effects of priming for the deaf signers. There was a significant main effect of priming between 400–600 ms (F(1,19) = 18.77, *p* = .0004, $\eta_{p}^{2}$ = .0658), between 600–800 ms (F(1,19) = 59.01, *p* < .0001, $\eta_{p}^{2}$ = .1698), between 800–1000 ms (F(1,19) = 29.61, *p* < .0001, $\eta_{p}^{2}$ = .1281), and between 1000–1400 ms (F(1,19) = 10.73, *p* = .004, $\eta_{p}^{2}$ = .0403).

### Time-course analysis

In order to examine the onset of priming effects (match vs. no-match), a time-course analysis using a sequence of paired t-tests for 50 ms epochs from 400–800 ms was run for the hearing learners after learning and for the deaf signers. Recall that the average sign onset was 578 ms after the start of the video. *P*-values reported in Table 5 are after FDR correction.

## Discussion

As expected, highly iconic signs were learned faster and more easily than non-iconic signs. After just one learning session, participants provided the correct English translations for 93% of the iconic ASL signs, compared to only 62% of the non-iconic signs (Table 2). In addition, before any learning took place, participants were able to guess whether or not the meaning of an English word matched the meaning of an iconic sign with high accuracy (96%), but guessing was just above chance for non-iconic signs (64%). Essentially, little learning was needed for these highly iconic signs as there was no change in translation accuracy for pre- vs. post-learning, although translation decision times were faster after learning, indicating that iconic signs were recognized faster with practice. For non-iconic signs, translation decisions were more accurate (98% vs. 64%) and much faster (1299 ms vs. 1820ms) after learning. Accuracy improved across sessions more for translation equivalents (matches; 39% vs. 97%) compared to mismatches (89% vs. 99%). This pattern is likely due to a strong tendency for participants to respond “no match” for the non-iconic signs prior to learning (Figure 2A) because (by design) these signs did not have a transparent meaning.

In contrast to the learners, translation decisions for deaf signers did not differ for iconic and non-iconic signs in either speed or accuracy, replicating Mott et al. (2020). Match decisions were faster than no-match decisions for the signers, following the typical pattern (“no” responses tend to take longer than “yes” responses in this type of task).

The neural changes that occurred with learning also differed between non-iconic and iconic signs. For non-iconic signs prior to learning, there was no neural evidence that participants had associated any meaning with these signs, as there was little difference between match and no-match trials, which indicates no priming effect from the English word to the ASL sign (Figure 3B). However, after learning, priming (larger negativities for no-match than match trials) was observed for the first three epochs (Figure 4B). This result is consistent with the behavioral results indicating that the non-iconic signs had become meaningful for the learners. In contrast, for iconic signs, large priming effects were observed before any learning occurred (Figure 3A). This result is consistent with the behavioral results and indicates that the meanings of these iconic signs were transparent to non-signers.

After the learning sessions, the priming effect decreased for iconic signs, and this reduction was most evident in the later epochs (Figures 3A and 4A). This finding suggests that the N400 priming effect for iconic signs is strongest before learning and decreases as the signs become more familiar. However, this pattern could also be partially explained as a change in the P3 (Late Positive Component) given that the N400 and P3 components can overlap in time. Specifically, before learning, the iconic match trials were the only trials in which the ASL sign could be recognized as the correct English translation, and these trials constituted only 25% of all trials. Thus, the iconic match trials might have acted like oddball events. If so, iconic signs may have elicited a larger P3 (LPC) amplitude before learning compared to after learning when all signs would have meaning and 50% of them would be recognized as matching the English word, eliminating any “oddball effect” from the data. A P3 effect was not observed for the non-iconic signs under similar circumstances (i.e., before learning, in the match condition) because most of them were not recognized as matching the prime, and therefore fell into the “standard” categoiy.

For the deaf ASL signers, there is no expectation of a P3 effect since all signs were known, and thus the percentage of match and no-match trials were equal. The direction of the priming effect (greater negativity for no-match than match trials) was the same for iconic and non-iconic signs, and its onset earlier for the deaf signers than for the learners (Table 5). Both findings replicate Mott et al. (2020).

The emergence of lexico-semantic priming effects for the non-iconic signs after learning is consistent with results from spoken L2 learning. For example, McLaughlin et al. (2004) found that the size of the N400 priming effect for L2 French learners (smaller N400 amplitudes to French words preceded by semantically related versus unrelated words) increased with learning. Pu et al. (2016) reported that the translation priming effect for L2 Spanish learners increased with only four hours of instruction (i.e., decreased N400 amplitudes for English words preceded by the Spanish translation versus an unrelated Spanish word). The spoken words learned in these studies were not iconic (i.e., onomatopoeic words were not included). Our parallel findings with non-iconic signs suggest similar neural learning processes occur when L2 vocabulary is learned in a new modality. Further, the fact that there is no phonological or orthographic overlap between ASL signs and their English translations indicates that priming occurs via lexical-level semantic connections created through vocabulary learning.

Although our results showed no priming effects for the non-iconic signs before learning, there were some trials where participants were able to correctly identify some of the non-iconic signs. To investigate whether correct responses to non-iconic signs patterned like iconic signs, we ran a post-hoc analysis on these trials (correct match trials (39%) and correct no-match trials (89%) for non-iconic signs). The results revealed parallel findings for correctly identified non-iconic signs, as evidenced by a significant interaction from 800–1000 ms between Match and Anteriority (*p* = .0126), as well as a significant main effect of Match (*p* = .001) and an interaction between Match and Laterality (*p* = .0331) in the 1000–1400 ms time-window (full statistical results and ERP waveforms and voltage maps are provided in the Supplementary Materials). These results indicate that the English translation prime provided enough information for participants to attach meaning to some non-iconic signs even before learning. For example, although the sign BEHIND is rated as non-iconic (2.63) and non-transparent (3.31), there is an iconic mapping such that one hand moves behind the other hand. Given the English translation, participants may have been able to attribute more meaning to BEHIND in the match condition compared to the no-match condition with the prime word “price”. The before learning priming effect for correctly identified non-iconic signs occurred later (800–1000 ms) compared to iconic signs (600–800 ms), likely because learners may have taken longer to ascertain the form-meaning mapping for these non-iconic signs. After learning, the timing of the priming effect was similar for iconic and non-iconic signs, suggesting that both sign types had a similar time course for lexical retrieval. The fact that some non-iconic signs were correctly identified highlights the idea that iconicity is a continuum (not all or none) and is subjective (can vary by individual) (Occhino et al., 2017; Sehyr & Emmorey, 2019).

The N400 priming effect that we observed prior to learning for iconic signs contrasts somewhat with the results of Ortega et al. (2020) who found no modulation of the N400 amplitude for iconic NGT signs prior to learning. However, there are several methodological differences that could account for the different results. First, Ortega et al. (2020) did not present no-match (unrelated) trials; rather, all prime-target pairs were translation equivalents, and the degree of gestural overlap (high versus low) was manipulated. Given that all pairs matched in meaning, it is perhaps not surprising that N400 effects were not observed. Second, all NGT signs were iconic, whereas both iconic and non-iconic signs were included here, which may have enhanced participants’ sensitivity to the form-meaning overlap for iconic signs. Third, participants in the Ortega et al. study passively viewed the Dutch-NGT prime-target pairs, whereas participants in our study made overt translation decisions on each trial. The differences in task and stimuli could account for why Ortega et al. found P3 modulations (larger P3 amplitudes for low-overlap signs which violated gesture expectations) whereas we found N400 modulations (smaller negativities for iconic signs preceded by translations that overlapped in meaning).

Ortega et al. (2020) found that the P3 difference between signs with high versus low gesture overlap disappeared after learning, supporting their interpretation that the before-learning P3 effect reflected expectation violations. We found that the N400 priming effect for iconic signs remained after learning, but the amplitude of the effect decreased. We interpret this change as a type of repetition effect. Specifically, the size of lexico-semantic priming is known to diminish with repeated exposure and increased predictability (see Kutas & Federmeier, 2011, for review). The reduced N400 priming effect further suggests that the effect of iconicity on lexical-semantic processing diminishes with learning and proficiency. An interesting avenue for future research would be to investigate at what point (if any) does the effect of iconicity disappear such that L2 learners resemble deaf signers and show no behavioral or ERP iconicity effects on translation decisions.

Iconicity may play a larger role in learning than in fluent language processing, as evidenced by the lack of an iconicity effect for the deaf signers, compared to the learners. For fluent signers, effects of iconicity appear to be highly task dependent. For example, iconicity can facilitate processing in some tasks (e.g., picture naming; McGarry et al., 2021; Navarrete et al., 2017), slow processing in others (e.g., phonological decisions: Thompson et al., 2010), and has no impact in some tasks (e.g., lexical decision: Bosworth & Emmorey, 2010; translation tasks: Gimeno-Martínez & Baus, 2022; McGarry et al., 2023). During language processing, iconicity may only come into play if it is task relevant in some way, e.g., there is alignment between visual features of a picture and the form of a sign which facilitates lexical retrieval (McGarry et al., 2021, 2023) or sign recognition (Thompson et al., 2009; Vinson et al., 2015). For lexical decision, the word-sign matching task used here, and translation tasks, the iconic form of signs is not relevant to the task (see also Emmorey, 2014). For adult learners, iconicity is generally beneficial for acquiring the meanings of signs because a) iconic signs constitute “manual cognates” with gestures (Ortega et al., 2020) which facilitates learning and b) adults can use form-meaning mappings as a mnemonic tool. Once signs are fully acquired and integrated into a large lexicon, the role of iconicity may be reduced, and other factors such as lexical frequency have a larger impact on processing (Caselli et al., 2021; Emmorey et al., 2020).

The timing and morphology of the N400 priming effect is impacted by the dynamic nature of signs and the fact that full videos were used in this experiment. Specifically, the videos were not edited to remove transitions to sign onset from a resting position, and ERPs were time-locked to video onset. Emmorey et al. (2022) showed that when ERPs are time-locked to video onset, the variability in sign onsets within the video leads to N400 priming effects that have an extended duration, in contrast to when videos are edited to remove transitional information. In the current study, sign onsets within the videos were indeed variable, with an average standard deviation of 104 ms. The average sign onset was 578 ms, and priming effects were observed in the 400–600 ms time window for both types of signs after learning and for both learners and deaf signers. This finding suggests that both learners and proficient signers were able to take advantage of cues within the transition to sign onset to constrain sign recognition. However, our time course analysis (Table 5) revealed that the deaf signers were able to take advantage of these cues earlier than the L2 learners. Note that Emmorey et al. (2022) reported that the duration of the N400 priming effect did not differ between time-locking to video onset and time-locking to sign onset within the video.

In sum, the effect of learning on N400 priming effects was reversed for non-iconic and iconic signs. After learning, the size of the priming effect increased for non-iconic signs but decreased for highly iconic signs. For deaf signers, iconicity did not modulate the size of the N400 priming effect. Overall, we conclude that iconicity impacts the time course of L2 sign language acquisition, and this effect is reduced over time when both iconic and non-iconic signs are learned as part of an emerging lexicon in the visual-manual modality.

## Supplementary Material

Supplementary Materials

## Figures and Tables

**Table 1.** Means and standard deviations for the descriptive characteristics of the iconic and non-iconic signs. *p*-values reported beneath each comparison reflect the t-test results for the relevant comparison between the iconic and non-iconic signs.

	Concreteness M (SD)	Word Frequency M (SD)	Sign Frequency M (SD)	Iconicity M (SD)	Transparency M (SD)	Duration M (SD)	Sign Onset M (SD)
**Iconic**	3.96 (.84)	4.52 (.74)	4.16 (1.16)	6.31 (.51)	5.05 (.60)	2189ms (331ms)	569ms (97ms)
**Non-Iconic**	3.82 (.89)	4.40 (.65)	4.15 (1.14)	1.92 (.47)	3.37 (.34)	2124ms (242ms)	587ms (111ms)
	*p* = 0.4207	*p* = .3863	*p* = .9869	*p* < .0001	*p* < .0001	*p* = .2696	*p* = .4064

**Table 2.** Mean (M) accuracy and standard deviations (SD) for all four testing sessions.

Test Session	% Total correct M (SD)	% Iconic correct M (SD)	% Non-Iconic correct M (SD)
After 1^st^ session	77% (10.77%)	93% (6.37%)	62% (15.17%)
Prior to 2^nd^ session	74% (18.21%)	88% (16.94%)	57% (19.48%)
After 2^nd^ session	93% (18.47%)	95% (17.62%)	86% (19.31%)
After 2^nd^ ERP session	96% (4.82%)	99% (1.89%)	94% (7.74%)

**Table 3.** Means (M) and standard deviations (SD) for accuracy and reaction times for the word-sign matching task for the hearing learners. Note that 50% accuracy represents chance.

	RT	Accuracy
Before learning iconic	1402ms (243ms)	96% (3.5%)
After learning iconic	1178ms (165ms)	99% (1.5%)
Before learning non-iconic	1820ms (344ms)	64% (11.5%)
After learning non-iconic	1299ms (190ms)	98% (3%)

**Table 4.** Means and standard deviation for reaction time and accuracy for the deaf signers in the word-sign matching task.

	RT	Accuracy
**Iconic Match**	1155ms (200ms)	97% (2.5%)
**Iconic No-Match**	1195ms (210ms)	97% (3.7%)
**Non-Iconic Match**	1143ms (183ms)	97% (2.8%)
**Non-Iconic No-Match**	1182ms (218ms)	99% (1.5%)

**Table 5:** Time-course analysis of priming onset for hearing learners after learning and for deaf signers. (** p<.05, *** p<.01, FDR corrected).

	400-450	450-500	500-550	550-600	600-650	650-700	700-750	750-800
**Hearing Learners After Learning**				**	**	**	**	***
**Deaf Signers**	**	***	***	***	***	***	***	***

## Data Availability

The stimuli and data that support these findings are available at Open Science Framework at https://osf.io/vpzxs/

## Footnotes

1 By convention, ASL signs are written in uppercase using the closest English translation. All ASL signs that are used for examples are hyperlinked to the ASL LEX website.

2 For the deaf participants there were 47 non-iconic signs because three were removed, one had two possible English translations (“Atlanta” and “assembly”), one was very low frequency (SPACESHIP), and one had regional variations (KANGAROO).

3 d’ scores showed a similar pattern (See Supplementary Materials for complete d’ statistics). We chose to use percent correct in order to separately examine “yes” (match) and “no” (no-match) decisions.
