# Supplementary material for: Neural effects differ for learning highly iconic versus non-iconic signs in hearing adults
Source: Biling (Camb Engl). Author manuscript; Available in PMC 2024 Nov 7. (PMC11542927; doi:10.1017/s1366728923000809)
Supplement: Supplementary Materials [file NIHMS1947199-supplement-Supplementary_Materials.docx]

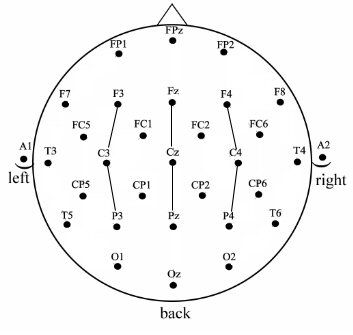


**Supplementary Figure 1**. Electrode montage and nine analysis sites (sites connected with lines)


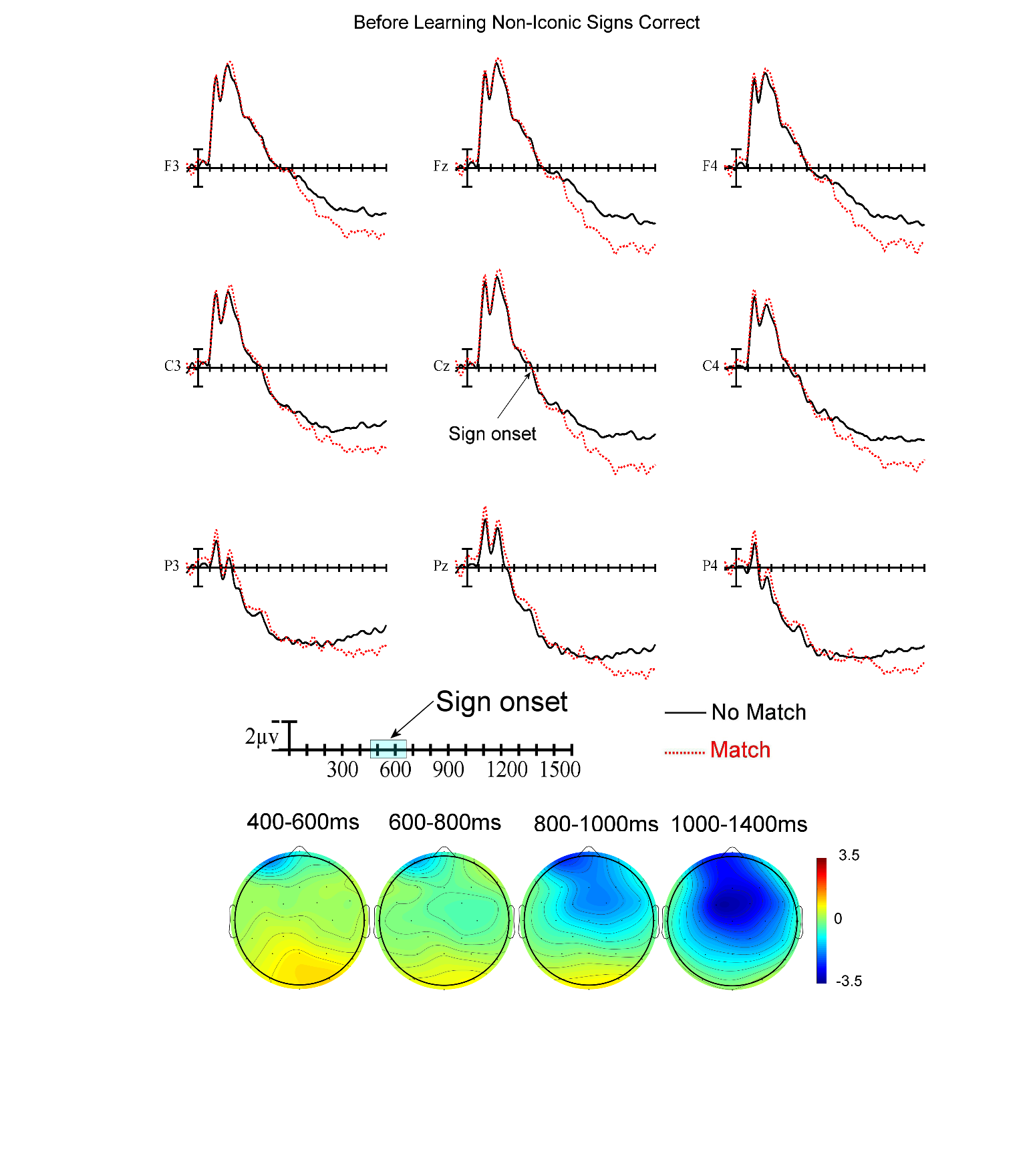


**Supplementary Figure 2.** Top) ERPs to non-iconic ASL signs before learning at the 9 electrode sites used in the ANOVAs. Bottom) Voltage maps formed by subtracting no-match trial ERPs from match trial ERPs in the four latency ranges. **Results:** There were no significant main effects (all *p*s > .41) from 400-600ms, 600-800ms, or 800-1000ms. There were also no significant interactions (all *p*s > .23) in the time-windows 400-600ms or 600-800ms. There was a significant interaction between Match and Anteriority between 800-1000ms (F(2,62) = 6.3, *p* = .0126, η_p_^2^ = .0025). In the 1000-1400ms time-window there was a main effect of Match (F(1,31) = 13.2, *p* = .001, η_p_^2^ = .0222) as well as a significant interaction between Match and Laterality (F(2,62) = 3.82, *p* = .0331, η_p_^2^ = .0005).

**D’ results**

There was a significant main effect of Learning (F(1,31) = 122.95, *p* < .001, ηp2 = .2955); main effect of Matching (F(1,31) = 32.3, *p* < .001, ηp2 = .0476); main effect of Iconicity (F(1,31) = 131.6, *p* < .001, ηp2 = .0758). As well as significant interactions between Learning and Matching (F(1,31) = 11.55, *p* = .0019, ηp2 = .031); between Learning and Iconicity (F(1,31) = 131.6, *p* < .001, ηp2 = .0758); between Matching and Iconicity (F(1,31) = 23.82, *p* < .001, ηp2 = .0212); and between Learning, Matching and Iconicity (F(1,31) = 23.82, *p* < .001, ηp2 .0212).
